# Evaluation of a multi-arm multi-stage Bayesian design for phase II drug selection trials – an example in hemato-oncology

**DOI:** 10.1186/s12874-016-0166-7

**Published:** 2016-06-02

**Authors:** Louis Jacob, Maria Uvarova, Sandrine Boulet, Inva Begaj, Sylvie Chevret

**Affiliations:** 1 Biostatistics and Clinical Epidemiology team (ECSTRA), of the Center of Research on Epidemiology and Biostatistics Sorbonne Paris Cité (CRESS; INSERM UMR 1153), Paris Diderot University, SBIM- Hôpital Saint Louis; 1, av Claude Vellefaux 75010, Paris, France; 2 École Normale Supérieure de Lyon, 46 Allée d’Italie, 69007 Lyon, France; 3 École Nationale de la statistique et de l’analyse de l’information, Rue Blaise Pascal, Rennes, France

**Keywords:** Bayesian, MAMS, Drop/select drug, Adaptive design

## Abstract

**Background:**

Multi-Arm Multi-Stage designs aim at comparing several new treatments to a common reference, in order to select or drop any treatment arm to move forward when such evidence already exists based on interim analyses. We redesigned a Bayesian adaptive design initially proposed for dose-finding, focusing our interest in the comparison of multiple experimental drugs to a control on a binary criterion measure.

**Methods:**

We redesigned a phase II clinical trial that randomly allocates patients across three (one control and two experimental) treatment arms to assess dropping decision rules. We were interested in dropping any arm due to futility, either based on historical control rate (first rule) or comparison across arms (second rule), and in stopping experimental arm due to its ability to reach a sufficient response rate (third rule), using the difference of response probabilities in Bayes binomial trials between the treated and control as a measure of treatment benefit. Simulations were then conducted to investigate the decision operating characteristics under a variety of plausible scenarios, as a function of the decision thresholds.

**Results:**

Our findings suggest that one experimental treatment was less efficient than the control and could have been dropped from the trial based on a sample of approximately 20 instead of 40 patients. In the simulation study, stopping decisions were reached sooner for the first rule than for the second rule, with close mean estimates of response rates and small bias. According to the decision threshold, the mean sample size to detect the required 0.15 absolute benefit ranged from 63 to 70 (rule 3) with false negative rates of less than 2 % (rule 1) up to 6 % (rule 2). In contrast, detecting a 0.15 inferiority in response rates required a sample size ranging on average from 23 to 35 (rules 1 and 2, respectively) with a false positive rate ranging from 3.6 to 0.6 % (rule 3).

**Conclusion:**

Adaptive trial design is a good way to improve clinical trials. It allows removing ineffective drugs and reducing the trial sample size, while maintaining unbiased estimates. Decision thresholds can be set according to predefined fixed error decision rates.

**Trial registration:**

ClinicalTrials.gov Identifier: NCT01342692.

## Background

Adaptive designs for clinical trials that use features that change or “adapt” in response to information generated during the trial to be more efficient than standard approaches [1] have been the focus of an abundant statistical literature since the 1970s. Among the wide range of adaptive designs, multi-arm multi-stage (MAMS) designs aim to compare several new treatments (multi-arm) to a common reference treatment to select or drop any treatment arm to move forward when evidence exists based on interim analyses (multi-stage). These designs have also been referred to as selection designs in phase II/III trials [2], randomized phase II screening trials [3] or select-drop designs [4]. Similarly to other adaptive designs, MAMS designs aim to decrease the time and number of patients required to move experimental treatments from development to a definitive assessment of benefit compared to the traditional approach, in which each drug is assessed through separate controlled trials. Improving the efficiency of clinical trials has been of prime interest in the development of anticancer therapies because multiple candidate anticancer agents are available for screening simultaneously due to the acceleration of drug development [3, 5]. However, although MAMS trials have gained popularity, they are still poorly used by practitioners. Notably, because of the number of arms and stages, MAMS trials appear more complex in design, conduct, and data analysis, with a broad variety of proposed versions [6–8]. All these proposed MAMS trials are faced with the issue of multiple testing due to comparisons between active treatments and control treatment, or pairwise between all arms. Moreover, this multiplicity issue is increased by the repeated testing, resulting in stopping either the trial or merely the relevant arm, with a focus on sequential futility boundaries for lack of benefit adjusted so that the overall familywise error rate is or is not controlled at a pre-specified α level.

We aimed at assessing how a Bayesian MAMS design may appear as an alternate way of handling such issues. Indeed, Bayesian designs are an efficient way to achieve valid and reliable evidence in clinical trials, given that the interpretation of the data is unrelated to preplanned stopping rules and is independent of the number of interim views [9, 10]. Such Bayesian approaches for MAMS trials have been rarely used, notably with one proposal for normal outcomes [11]. To allow a direct and simple use of the Bayes approach, we focused on the probability of success in binomial trials, restricting our considerations to conjugate beta priors. Moreover, it can then be easily updated along the trial, and allowance for early stopping for futility can be made. This setting of Bayes binomial trials was also recently used to compare the Bayesian approaches to frequentist hypothesis testing in two-arm clinical trials [12]. Actually, our approach could be also viewed as an extension to the MAMS trials with binary outcomes of that proposed by Zalavsky for two-arm trials [12]. Indeed, both approaches use similar beta-binomial modeling (with integers [12] or not as beta parameters), and posterior difference of beta as the quantity of interest for decision making. However, while Zalavsky [12] focused on deriving one-sided superiority and non-inferiority Bayesian tests and their closeness to frequentist approaches, we provided stopping rules as decision-tools for interim analyses due to the MAMS design, as Xie et al. did [13]. The scope for extending this approach to the comparison of different arms of experimental treatments against one control was considered below.

This paper was motivated by a phase II randomized controlled trial to compare on a binary outcome measure, two experimental drugs with conventional azacitidine treatment for myelodysplastic syndrome patients, in which the main objective was to drop the experimental inefficacious arm. The trial was designed using a modified two-stage Simon’s design [14], allowing with small sample sizes of 40 patients per arm in the first stage to control the type I error accurately at the pre-specified level of 0.15 with a statistical power of 0.80. At the end of this first stage, no decision of dropping any arm was made. We wondered whether the use of a Bayes approach may have modified the design, and subsequent analyses.

Thus, the objective of this paper was to redesign the Bayesian adaptive design originally proposed by Xie, Ji and Tremmel for seamless phase I/II trials [13], focusing on the comparison of multiple experimental drugs to a control drug on a binary criterion measure.

First, we applied our design to the real dataset from the ongoing phase II randomized trial conducted on 120 patients that motivated this work. Then, we assessed its performance using a simulation study. Some discussion and conclusions are finally provided.

## Methods

### Motivating example

We used data from a phase II clinical trial of an international study conducted in 120 patients with myelodysplastic syndrome (MDS) who were randomized across three treatment arms. Although the original design was non-Bayesian [14], we reanalyzed data from the first stage of this trial to illustrate the interest of Bayes approaches. Because the trial is still ongoing in a second stage, no further details about the treatment arms will be provided. Each group of 40 patients received one of the following treatments: A (reference treatment, control group), B or C (two combinations of new drugs with the reference treatment, experimental groups). It was hypothesized that the response rate in the control group would be 0.30 and that a response rate of at least 0.45 would indicate that a combination was sufficiently promising to be included in further studies.

### Bayesian multi-Arm multi-stage design

Let X denote the treatment arm, where X = 0 is the control arm, and X = 1, …, K denote K distinct new drugs to be tested. Suppose that *n* patients are randomly allocated to each of the (K + 1) arms. For simplicity, let us consider a balanced design, although any imbalanced fixed design could be considered.

Consider a binary outcome, Y, where Y = 1 denotes a response to treatment and Y = 0 denotes the absence of a response. The observed number of responses among the ${n}_k$ patients allocated to arm *k* is given by ${y}_k=\sum_{i=1}^n{y}_i{1}_{i\in k}$, where ${1}_{i\in k}$ denotes the indicator function (${1}_{i\in k}=1$ if the ith patient has been allocated to arm k, and 0 otherwise). Note that the selection does not need to involve a measure of efficacy [2], so that response could be defined according to a toxicity grading scale.

We used a Bayesian inference framework, where ${\pi}_k=P\left(Y=1|X=k\right)$ denotes the probability of response in the arm X = k (k = 0, …, K). Using a beta Be(${a}_k$, ${b}_k$) prior for π_k_, the posterior probability of π_k_ is still a beta distribution given by Be(${a}_k$ + ${y}_k$, ${b}_k$ + ${n}_k-{y}_k$) due to the natural conjugate property of the beta family for binomial sampling.

The main aims of MAMS trials are to, over a range of K new treatments, select those that prove sufficiently efficacious and avoid those drugs that are unexpectedly ineffective. Let ${y}_{ki}$ denote the number of responses observed at stage *i* among the ${n}_{ki}$ patients randomly allocated to arm X = *k* (k = 0, …, K).

Thus, several stopping decision criteria were proposed, derived from the proposals of Xie [13].

First, the inefficacy of each drug was assessed by comparison to some historical minimal value of interest, which was originally called the “minimum required treatment response rate” (MRT) by Xie et al. [13]. Thus, the futility rule (denoted as Rule 2 in [13]) is defined by the following posterior probability:

1 $$ P\left({\pi}_k<{p}_0\Big|{y}_{ki},{n}_{ki}\right)>{\gamma}_1 $$

where ${p}_0$ denotes the MRT usually defined from some historical control rates, and ${\gamma}_1$ is some threshold of a “high” probability of inefficacy.

In randomized phase II settings, the selection of a new drug is based on evaluating the potential benefits of the experimental treatment over the control arm [15]. Thus, one may consider dropping a new drug from further studies only if there is a rather low posterior probability that this drug is beneficial over the control by some targeted minimal level while on the opposite selecting the drug if there is sufficient information to declare that one treatment is better than the other, that is when its benefit reaches a so-called “sufficient treatment response rate” (STR). Two resulting decision criteria and stopping rules were defined from the posterior distribution of the difference in response rates of the experimental over the control arm at the ith stage as follows:

2 $$ P\left({\pi}_k-{\pi}_0>\triangle\ \Big|{y}_{ki},{n}_{ki}\right)<{\gamma}_2 $$

3 $$ P\left({\pi}_k-{\pi}_0>{\delta}^{*}\Big|{y}_{ki},{n}_{ki}\right)\kern0.5em >{\gamma}_3 $$

In the original paper [13], Eq. (2) is referred to as Rule 3, with $\Delta$ set at the “targeted difference in response rate”, and Eq. (3) is referred to as Rule 4, with ${\delta}^{*}$ set at the STR. However, whereas Xie [13] used the Eq. (2) to define expansion for the seamless phase I/II design, in the present study, we only considered select/drop decisions due to the phase II design. More specifically, Eq. (2) attempts to assess the futility of experiencing experimental arm k given the posterior probability that its response rate compared to that observed in the control arm is below some decision threshold; such a rule (2) can be considered as the posterior probability of the alternative hypothesis, as commonly used to evaluate the success of an experiment. Thus, such a rule was proposed to provide an answer closest to the frequentist setting where one wishes to test the null against the alternative. Note that when Δ = 0, the equation (2) reduces to the posterior probability that the experimental treatment is better than the control, a quantity that was first proposed in the setting of phase 2 single arm clinical trials [15] and more recently used to provide adaptive randomized allocation probability [16, 17]. By contrast, Eq. (3) aims at quantifying the posterior probability that response rate in experimental arm k is above that of the control arm by some sufficient treatment response rate. From a practical perspective, the alternative hypothesis in terms of differences in response rates that aim for better performance (or non-inferiority) could be considered, and appear very natural in the clinical environment.

Contrary to the posterior density given in (1), the second and third rules involve the difference of two beta distributions (${\pi}_k$ and ${\pi}_0$, respectively), which is no longer a beta distribution but a complicated distribution as reported in [12]. This difference has been computed in relation to Appell’s hypergeometric functions [18, 19]; otherwise, a normal approximation has been proposed; however, when the difference between the sample proportions is small, the approximate probability is not equal to the exact probability [19]. Exact calculation is possible in a few special cases [20], while numerical integration is usually performed, like in [12, 15]:

4 $$ P\left({\pi}_k<{\pi}_0+d\left|{y}_k{n}_k{y}_0{n}_0\right.\right)={\displaystyle \underset{0}{\overset{p-d}{\int }}}F\left({\pi}_k+d\Big|{a}_k+{y}_k,\ {b}_k+{n}_k-{y}_k\right) \times f\left(p\Big|{a}_0+{y}_0,\ {b}_0+{n}_0-{y}_0\right)dp $$

where F(|a,b) and f(|a,b) are the cumulative distribution function and the density of the beta random variable π ~ Be(a,b), respectively.

### The priors

Regarding the prior on the response probability, ${\uppi}_{\mathrm{k}},\;\mathrm{k}=0, \dots, \mathrm{K}$, the amount of past information is likely different according to the randomization arm. While it is expected that the elicitation of the prior on ${\uppi}_0$ could be based on previous trial results and expert opinion, that on ${\uppi}_{\mathrm{k}},\;k>0$, is likely to be less informative.

First, the use of flat non-informative priors was motivated by several considerations. It allows the posterior to be dominated by the data rather than by any prior overoptimistic views regarding the experimental arms. Thus, it insures that critical amount of clinical information is required as a basis for deciding whether the experimental arm will be administered to a large number of patients in a Phase III clinical trial. Moreover, such domination by the data allows the trial results to be used by others who have their own priors [15].

**Fig. 1:**
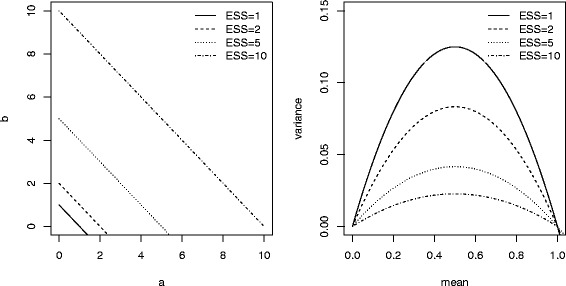
Guide calibration of the prior variance according to the prior mean and the prior information translated into the so-called effective sample size (ESS) – For instance, when prior mean is 0.50, the variance is 0.125, 0.083, 0.042, and 0.027 for a prior effective sample size of 1, 2, 5 and 10, respectively

However, it is widely recommended to use different prior densities to assess the robustness of the trial results. Thus, we performed sensitivity analyses to the prior choice, using distinct beta distributions reflecting increased amount of prior information throughout the effective sample size (ESS) [21]. Given the ESS of a beta Be(a,b) prior is given by ESS=a+b, one may modifying the beta parameters for modifying the prior variance while the prior mean is fixed, providing sensitivity analyses to the prior information translated into a sample size (Fig. 1). Prior mean was either “enthusiastic” or “skeptical”, as we did previously [22]. These terms “enthusiastic” and “skeptical” priors refer to either the optimistic view of a beneficial treatment effect at least equal to that expected when planning the trial, or to the pessimistic view of no treatment effect as compared to the control [23]. Both priors allow encompassing the heterogeneity in physician prior opinion before to the trial.

### Decision thresholds

To be applied, some arbitrary constants (further denoted as “design parameters”) must be defined. First, the choice of the minimal response rate (*p*_*0*_) could be guided by some historical controls or the clinical experience of the control group in the disease under study, and the response rate under the null hypothesis is commonly chosen in uncontrolled Phase II trials. Second, we choose $\Delta =0$ as a targeted minimal response rate; this value represents no difference between the treatments. ${\delta}^{*}=0.15$ was chosen as a sufficient response rate; this value would reflect a clinically important treatment effect. Both values delineate the underlying null and alternative hypotheses in a frequentist framework.

Otherwise, the number of design stages, that is, the frequency of the computation of the rules described above that conduct stopping decisions, should be defined. Moreover, the threshold values ${\gamma}_1,\;{\gamma}_2$ and ${\gamma}_3$ are statistical quantities that should be set to some predetermined values allowing for the good performance of the design, likely related to the quantity of information in the trial (thus, of the entire sample size). Xie in 2012 [13] suggested that ${\gamma}_1$ and ${\gamma}_3$ should be high (>0.8), and ${\gamma}_2$ should be at most 0.10. Obviously, such values widely govern the occurrence of false positive (or negative) decisions. Nevertheless, larger than traditional values of false positive rates are commonly used in MAMS settings, up to 0.50 at the first stage [8], notably because one wishes to make decision on dropping arms early while maintaining a low false negative decision rate.

Thus, we first proposed to compute the decision rules after every observed response in the trial and then attempt to define some criteria for design choices, and their impact in terms of sample size.

## Results

### Illustrative case study

We first apply the proposed design to the phase II randomized trial with K=2 new drugs compared against the control. The Jung trial design [14] was based on *p*_*0*_=0.30 and δ=0.15, with type I and type II errors fixed at 0.15 and 0.20, respectively. Of the 120 enrolled patients, 44 (36.7 %) exhibited a response, including 15 in arm A, 13 in arm B, and 16 in arm C, resulting in observed response rates of 0.3750, 0.3250 and 0.40, respectively.

Bayes analyses were applied, first using in each arm non-informative beta priors either the Jeffreys prior Be(1/2,1/2) or the uniform prior Be(1,1) resulting in ESS=1 or 2, respectively. Then, as reported above, a sensitivity analysis to the prior choice was performed; for the control arm, only skeptical priors - centered on the null (prior mean=0.3) hypothesis- were used, while both skeptical and enthusiastic – centered on the alternative (prior mean=0.45) hypothesis- priors were defined. Prior effective sample size was set at 10 in control, and varied from 1 or 5 in experimental arms.

**Fig. 2:**
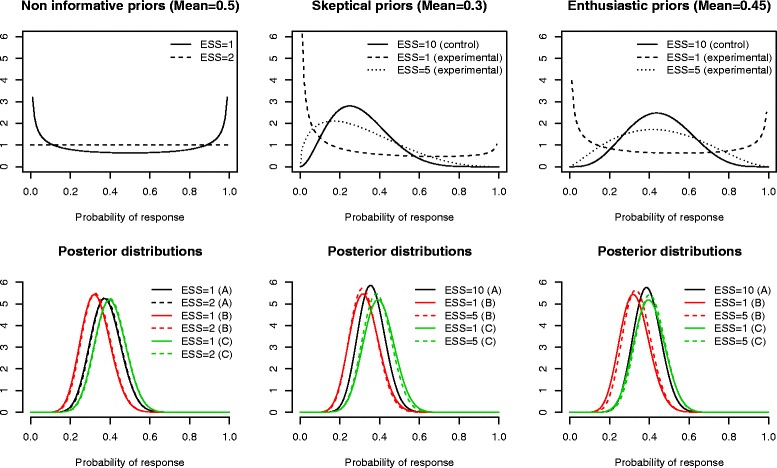
MDS trial- Sensitivity analyses of the distribution of response rate in each treatment arm according to the prior choice in terms of location (non-informative centered on 0.5, skeptical centered on 0.3 or enthusiastic centered on 0.45) and effective sample size (ESS ranging from 1–5 in experimental arms up to 10 in control). Upper plots display the prior densities while lower plots display the posterior densities. The left plots refer to the non-informative situation in which all of the three priors are uniform over [0,1] (Be (1,1)) or distributed according to Jeffreys prior (Be (1/2,1/2); the middle and right plots refer to the situations in which the priors were either skeptical (middle plots) or enthusiastic (right plots); each uses various effective sample sizes (ESS) denoting various amounts of prior information

Figure 2 displays the prior and posterior distribution of response rates in each randomized arm at the end of enrollment, illustrating how the posterior distribution of each experimental arm was not markedly affected by the prior information as translated into the (prior) effective sample size or its location. At the end of the trial, according to the prior, the posterior mean response rate ranged from 0.3600 to 0.3810 in arm A, from 0.3222 to 0.3389 in arm B, and from 0.3889 to 0.4056 in arm C (Table 1).

**Table 1:** MDS results – Sensitivity analyses

Prior	ESS			Posterior mean			Decision criteria						
Arm	A	B	C	A	B	C	A	B	C	B	C	B	C
							Rule 1	Rule 1	Rule 1	Rule 2	Rule 2	Rule 3	Rule 3
MLE	0	0	0	0.3750	0.3250	0.4000							
Non informative	1	1	1	0.3780	0.3293	0.4024	0.1505	0.3576	0.0863	0.3198	0.5906	0.0286	0.1197
	2	2	2	0.3810	0.3333	0.4048	0.1384	0.3346	0.0789	0.3223	0.5894	0.0281	0.1161
Sk eptical	10	1	1	0.3600	0.3244	0.3976	0.1900	0.3833	0.0971	0.3575	0.6437	0.0310	0.1340
	10	5	1	0.3600	0.3222	0.3976	0.1900	0.3885	0.0971	0.3465	0.6437	0.0262	0.1340
	10	1	5	0.3600	0.3244	0.3889	0.1900	0.3833	0.1074	0.3575	0.6148	0.0310	0.1099
Enthusiastic	10	1	1	0.3600	0.3222	0.3889	0.1900	0.3885	0.1074	0.3465	0.6148	0.0262	0.1099
	10	5	1	0.3600	0.3280	0.4012	0.1900	0.3640	0.0889	0.3716	0.6570	0.0338	0.1422
	10	1	5	0.3600	0.3389	0.4012	0.1900	0.2996	0.0889	0.4128	0.6570	0.0393	0.1422

The first line refers to the maximum likelihood estimate of response probability of each treatment arm, while the other lines refer to Bayes posterior estimates with computed decision criteria based on different combinations of the priors

*MLE* maximum likelihood estimate, *ESS* effective sample size, Decision criteria use p_0_=0.3, Δ=0, δ*=0.15: p_0_ refers to the minimum required treatment response rate of the first rule (Eq. 1); Δ to the targeted difference of the second rule (Eq. 2), and δ* to the sufficient treatment response rate of the third rule (Eq. 3)

**Fig. 3:**
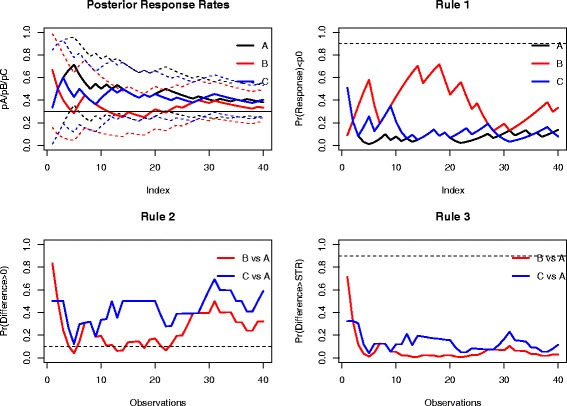
Results of the MDS trial- Bayesian analyses using non-informative uniform priors, the minimum required treatment response rate at MRT=0.3 (Rule 1), the targeted minimal response rate at Δ=0 (Rule 2), and a sufficient treatment response rate at STR=0.15 (Rule 3), with the cut-off probability thresholds for rules 1–3 set at 0.9, 0.1 and 0.9, respectively

We retrospectively applied the decision rules defined in (1)-(3) with threshold values set at 0.9, 0.1 and 0.9, respectively. Figure 3 displays the evolution of the posterior probabilities and stopping criteria over time, when using non-informative priors.

The application of the first stopping criterion does not allow either arm to be eliminated, indicating that there is a small probability that either response rate is below the historical response rate of 0.30; indeed, the posterior estimates were close to and mainly above 0.30, except for arm B, where the response rate was lower than those of the other two arms for the 20 first enrolled patients (Fig. 2, left). This finding was illustrated in the second criterion computed over the trial, where the cut-off threshold of the second decision criterion was crossed for arm B after 5, 13, 14, 18, and 22 enrolled patients in that arm, illustrating a low (<0.10) posterior probability that the response rate in that arm was above that observed in the control. As expected, the third decision criterion never required that the study be stopped with the conclusion that the benefit of any experimental arm was at least the 0.15 expected. Note that all the three decision criteria at the end of the trial were slightly affected by the prior, with close values that do not modify any decision (Table 1).

These findings suggest that arm B could have been dropped from the trial based on a sample of approximately 20 instead of the 40 actually recruited patients, although further results (with a sample size of at least 25 patients) do not confirm such a decision. This could be related to some “drift” towards improved response rates over the course of the trial. This may also point out that the probability in Eq. (2) can be highly variable in the beginning of the trial when the number of patients is small, resulting in possibly false decisions [17].

We thus decided to assess the performances of this approach and more specifically to assess the quantity of information required to drop an ineffective arm or an efficacious arm, according to decision thresholds related to false decision probabilities.

### Simulation study

#### Simulation settings

Once the Bayesian design has been structured, statisticians use simulations and adjust tuning parameters to comply with a set of targeted operating characteristics. Thus, we assessed the operating characteristics of the proposed MAMS design through simulations that mimic the MSD trial, although with clear-cut ineffective or effective drugs, and in which stopping decision criteria (1)-(3) were applied.

We considered several situations of drug inefficacy, that is, when the benefit in terms of response rate was null or below that expected of 0.15 (true benefit set at 0, 0.05, and 0.10 compared to an expected response rate of 0.30), and situations of drug efficacy (true benefit at 0.15, 0.20, 0.25, 0.30 and 0.45, over the 0.30 expected response rate). Moreover, among the K=2 new drugs, several scenarios combining these various treatment benefits were distinguished, either similar across new drugs or not. The first scenario simulated the case in which the efficacies of treatments B and C were similar to that of treatment A (${\pi}_B={\pi}_C={\pi}_A$). In further scenarios, we simulated the case in which only arm B was more efficient than A (${\pi}_C={\pi}_A,\;{\pi}_B={\pi}_A+ dB$). In the latter, we simulated the cases where both B and C treatments had a higher probability of response than A (${\pi}_C={\pi}_A+dC,\;{\pi}_B={\pi}_A+ dB$).

We simulated samples of $n$ patients. In each simulation, the treatment arm was generated from a multinomial distribution mult($n,\frac{1}{3},\frac{1}{3},\frac{1}{3}$), and the response-indicating efficacies were generated from Bernoulli distributions B(${\pi}_k$).

For each scenario, data were analyzed using Bayesian inference. The priors of ${\pi}_k$ were non-informative beta Be(1,1). Posterior probabilities in (1) were easily obtained as beta cumulative density functions, whereas those of (2) and (3) required numerical integration –see Eq. (4). We first computed those criteria for fixed sample sizes. Then, any arm could be dropped if evidence suggested that it was unlikely to be effective (futility rules 1 and 2) or if sufficient evidence of effectiveness over the control had already been determined (rule 3). Furthermore, to take into account the high variability in differences of beta distributions based on small samples [16], those rules only applied once at least 15 patients had been enrolled in each arm.

A total of *N*=10,000 independent replications were performed, with the results averaged across the N repeated simulations. In all simulations, design parameters were set to be constant at *p*_*0=*_0.30, $\mathrm{\triangle}=0$ and ${\delta}^{*}=0.15$ unless otherwise specified.

All analyses were performed using the R statistical software (http://www.R-project.org/).

#### Simulation results

### Threshold calibration

To determine the decision thresholds, as suggested by Xie [13], some simulations were first performed, considering a 2 fixed parallel arm designs based on *n*=40 and *n*=100 patients per arm (Table 2). In all cases, biases were low, mainly below 0.01 (when *n*=40) or 0.005 (when *n*=100), with lower mean square errors (MSEs). The first decision criterion, that is, the posterior probability that the response rate was lower than 0.3 was nearly equal to 0.5 in the control arm or when there was no drug benefit (dB=0), as expected, and then decreased from 0.30 (when dB=0.05 and *n*=40) down to 0.01 (when dB=0.25) to reach 0 when dB=0.45. In parallel, the difference between the probabilities of a response for B over the control arm A increased with the benefit of B. Moreover, a larger sample size led to a higher probability of detecting a smaller benefit, so that for a given benefit, the decision threshold depends on the amount of information.

**Table 2:** Simulation results in terms of absolute bias based on a fixed sample size for increasing benefit of the experimental arm– all priors on p_k_ (k=A,B,C) are non-informative Be (1,1) priors; p_0_=0.30; *n*=40 or 100 patients per arm

Sample size	True benefit	Posterior mean estimate biases		Mean square errors		Decision criterion 1		Criterion 2	Criterion 3
	dB	p_A_	p_B_	p_A_	p_B_	A	B	B	B
**40**	0.00	0.0086	0.0090	0.0048	0.0048	0.4850	0.4835	0.5012	0.1414
	0.05	0.0082	0.0078	0.0048	0.0052	0.4854	0.2982	0.6358	0.2399
	0.10	0.0100	0.0042	0.0050	0.0054	0.4798	0.1632	0.7440	0.3518
	0.15	0.0097	0.0014	0.0049	0.0055	0.4798	0.0753	0.8352	0.4803
	0.20	0.0098	−0.0001	0.0049	0.0057	0.4795	0.0297	0.9028	0.6132
	0.25	0.0096	−0.0013	0.0049	0.0056	0.4800	0.0093	0.9477	0.7322
	0.30	0.0095	−0.0049	0.0049	0.0055	0.4804	0.0027	0.9731	0.8271
	0.35	0.0088	−0.0070	0.0048	0.0051	0.4841	0.0005	0.9886	0.8999
	0.40	0.0109	−0.0087	0.0048	0.0047	0.4749	0.0001	0.9958	0.9477
	0.45	0.0092	−0.0116	0.0049	0.0043	0.4808	0.0000	0.9984	0.9755
**100**	0.00	0.0042	0.0032	0.0020	0.0020	0.4863	0.4925	0.4960	0.0460
	0.05	0.0037	0.0030	0.0020	0.0022	0.4885	0.2156	0.7042	0.1363
	0.10	0.0040	0.0014	0.0020	0.0023	0.4866	0.0661	0.8503	0.2896
	0.15	0.0038	0.0012	0.0020	0.0024	0.4881	0.0128	0.9404	0.4925
	0.20	0.0040	−0.0003	0.0020	0.0024	0.4858	0.0015	0.9807	0.6875
	0.25	0.0027	−0.0009	0.0020	0.0023	0.4947	0.0001	0.9950	0.8473
	0.30	0.0046	−0.0017	0.0020	0.0023	0.4836	0.0000	0.9989	0.9362
	0.35	0.0042	−0.0032	0.0020	0.0022	0.4856	0.0000	0.9998	0.9792
	0.40	0.0032	−0.0037	0.0020	0.0021	0.4922	0.0000	1.0000	0.9952
	0.45	0.0036	−0.0047	0.0020	0.0018	0.4897	0.0000	1.0000	0.9992

N is the sample size; p_A_, p_B_ and p_C_ refer to the posterior means of response probability in arms A, B and C, respectively; dB refers to the true benefit of B over A in terms of response probability. Bold data refer to the null hypothesis of absence of any treatment difference (dB=dC=0), or to the alternative hypothesis of an expected true 0.15 benefit of treatment B (dB=0.15)

**Fig. 4:**
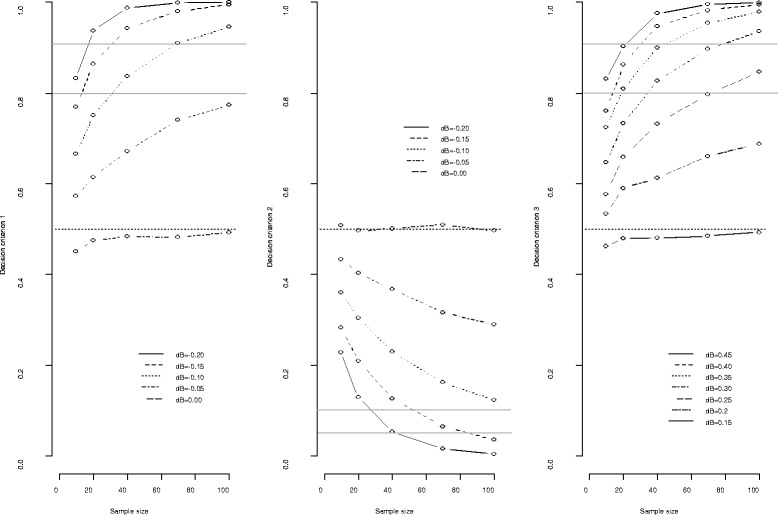
Posterior stopping rules according to the actual treatment benefit and sample size; the left plots refer to decision criterion 1 with p_0_=0.15, the middle plots refer to criterion 2 with Δ=0, and the right plots refer to criterion 3 with δ*=0.15. The mean estimates are from *N*=10,000 independent simulations for each actual benefit (dB)

We thus computed the three decision criteria according to the true benefit of the experimental arm (dB ranging from −0.2 to 0.45) and to the sample size (ranging from 10 to 100 patients per arm), each based on 10,000 independent replications (Fig. 4). Left plots of Fig. 4 quantify to what extent the stopping rule (1) is influenced by the sample size and the actual benefit of the experimental treatment arm – beside the threshold cut-off value, expectedly. Notably, it shows that a threshold of 0.95 with samples of *n*=40 patients per arm, allows on average arms with response rates below 0.15 of that expected to be dropped, while those with response rates below 0.10 could be dropped only when the sample size reached *n*=100. Similarly, when the experimental arm is compared to the control (middle plots), rule 2 evaluating the futility of trial continuation, with a 0.05 threshold, allows on average arms with a response probability at least 0.20 below that of control to be dropped when the sample size was *n*=40, and those with response probability 0.15 below to the control when *n*=100. In contrast, a threshold of 0.95 for rule 3 (right plots) will enable one to determine that the benefit of the experimental over the control is at least 0.40 with *n*=40, and nearly 0.30 with *n*=100.

Obviously, when the threshold values were less stringent, the increased ability of the design in dropping less different arms compared to the control could be counterbalanced by its increase propensity of dropping efficacious arms. This was the further aim of the simulation study to assess those false (positive or negative) decision rates.

### Assessing false decision rates

Tables 3, 4 and 5 summarize the simulation results for the arms dropped at the end of the first stage and the absolute bias in their treatment effect estimates on the definitive outcome at the stopping decision based on rules 1, 2 and 3, respectively, when the sample size was set at *n*=40, 100 per arm, and the threshold values were set at stringent values, that is, of ${\gamma}_1=0.95$, ${\gamma}_2=0.05$ and ${\gamma}_3=0.95.$

**Table 3:** Simulation results for dropping treatment arms based on the first rule (R1) and the absolute bias for such arms in the estimated treatment effect at the time of dropping decision– all priors on p_k_ (k=A,B,C) are non-informative beta(1, 1) priors, when decision threshold is set at 0.95

Sample size	True benefit		Posterior mean estimate biases			Enrolled sample sizes			% Early stopping		
	dB	dC	p_A_	p_B_	p_C_	n_A_	n_B_	n_C_	A	% early B	% early C
40	−0.20	0.00	−0.0051	0.0305	−0.0063	36.3504	15.0121	36.3180	15.32 %	96.09 %	15.15 %
	−0.15	0.00	−0.0058	0.0061	−0.0057	36.2548	21.3829	36.3305	15.43 %	79.69 %	15.19 %
	−0.10	0.00	−0.0063	−0.0066	−0.0062	36.3540	27.9657	36.3594	15.20 %	53.98 %	15.36 %
	−0.05	0.00	−0.0056	−0.0076	−0.0072	36.2719	33.0725	36.2428	15.44 %	30.66 %	15.86 %
	**0.00**	**0.00**	**−0.0050**	**−0.0057**	**−0.0063**	**36.2726**	**36.4040**	**36.4653**	**15.77 %**	**15.11 %**	**14.85 %**
	0.05	0.00	−0.0073	−0.0034	−0.0049	36.2420	38.1107	36.3738	15.75 %	7.33 %	15.23 %
	0.10	0.00	−0.0077	−0.0011	−0.0049	36.2214	39.1845	36.3761	15.91 %	2.92 %	14.88 %
	0.15	0.00	−0.0044	−0.0008	−0.0070	36.4279	39.6107	36.2580	14.86 %	1.35 %	15.47 %
	0.20	0.00	−0.0044	−0.0016	−0.0060	36.3945	39.8325	36.3640	15.06 %	0.55 %	15.29 %
100	−0.20	0.00	−0.0206	0.0305	−0.0205	84.2873	15.4339	84.3159	22.80 %	99.99 %	22.82 %
	−0.15	0.00	−0.0188	−0.0007	−0.0203	84.7484	24.5031	84.4050	21.95 %	99.27 %	22.22 %
	−0.10	0.00	−0.0202	−0.0212	−0.0197	84.4487	43.6230	84.5338	22.31 %	85.84 %	22.44 %
	−0.05	0.00	−0.0191	−0.0276	−0.0199	84.8712	66.7741	84.3454	21.99 %	52.46 %	22.57 %
	**0.00**	**0.00**	**−0.0216**	**−0.0207**	**−0.0216**	**84.0536**	**84.2135**	**83.8033**	**22.86 %**	**22.67 %**	**23.36 %**
	0.05	0.00	−0.0203	−0.0115	−0.0196	84.6003	93.1786	84.7271	22.28 %	8.66 %	22.24 %
	0.10	0.00	−0.0194	−0.0050	−0.0202	84.6521	97.3946	84.1845	21.94 %	3.08 %	22.76 %
	0.15	0.00	−0.0194	−0.0023	−0.0202	84.8085	98.9283	84.6060	22.00 %	1.20 %	22.06 %
	0.20	0.00	−0.0199	−0.0024	−0.0215	84.5757	99.4983	84.1044	22.02 %	0.56 %	22.94 %

p_A_, p_B_ and p_C_ refer to the posterior means of response probability in arms A, B and C, respectively; p_0_=0.3 (minimum required treatment response rate). Bold data refer to the null hypothesis of absence of any treatment difference (dB=dC=0), or to the alternative hypothesis of an expected true 0.15 benefit of treatment B (dB=0.15)

**Table 4:** Simulation results for dropping treatment arms based on the second rule (R2) and the absolute bias for such arms in the estimated treatment effect at the time of dropping decision – all priors on p_k_ (k=A,B,C) are non-informative beta(1,1) priors, when decision threshold is set at 0.05

Sample size	True benefit		Posterior mean estimate biases			Average sample sizes			% Early stopping	
n	dB	dC	p_A_	p_B_	p_C_	A	B	C	B	C
40	−0.20	0.00	0.0446	0.0410	−0.0036	34.7688	19.0005	34.1757	83.18 %	20.90 %
	−0.15	0.00	0.0407	0.0196	−0.0049	35.4230	23.9111	34.1465	65.96 %	21.12 %
	−0.10	0.00	0.0384	0.0076	−0.0045	36.0178	28.2528	33.9857	47.31 %	21.51 %
	−0.05	0.00	0.0325	0.0004	−0.0039	36.8988	31.7086	34.2757	31.84 %	20.67 %
	**0.00**	**0.00**	**0.0289**	**−0.0046**	**−0.0034**	**37.5750**	**34.3312**	**34.2628**	**20.68 %**	**20.55 %**
	0.05	0.00	0.0262	−0.0053	−0.0046	38.1404	35.9159	34.2368	13.61 %	21.01 %
	0.10	0.00	0.0212	−0.0068	−0.0038	38.6097	37.1804	34.2661	8.79 %	20.81 %
	0.15	0.00	0.0184	−0.0079	−0.0043	39.0075	38.0515	34.3098	5.79	20.69 %
	0.20	0.00	0.0163	−0.0098	−0.0051	39.2281	38.5864	34.2477	4.01 %	20.85 %
100	−0.20	0.00	0.0458	0.0362	−0.0144	80.7525	22.0750	80.0766	99.05 %	26.36 %
	−0.15	0.00	0.0427	0.0103	−0.0153	81.9307	34.6011	79.9595	91.50 %	26.58 %
	−0.10	0.00	0.0418	−0.0063	−0.0152	83.5055	51.2404	79.0897	71.48 %	27.30 %
	−0.05	0.00	0.0344	−0.0142	−0.0153	87.6493	67.8997	80.1142	46.17 %	26.49 %
	**0.00**	**0.00**	**0.0277**	**−0.0161**	**−0.0157**	**90.8189**	**79.7603**	**79.7672**	**26.62 %**	**26.82 %**
	0.05	0.00	0.0247	−0.0149	−0.0154	93.1423	86.5031	79.4970	15.96 %	26.65 %
	0.10	0.00	0.0202	−0.0130	−0.0157	95.3857	91.6455	78.7749	9.26 %	27.54 %
	0.15	0.00	0.0154	−0.0119	−0.0155	96.8064	94.2754	79.4074	6.15 %	27.04 %
	0.20	0.00	0.0112	−0.0100	−0.0147	97.8693	96.1853	80.1669	4.03 %	26.14 %

p_A_, p_B_ and p_C_ refer to the posterior means of response probability in arms A, B and C, respectively; Δ=0. Bold data refer to the null hypothesis of absence of any treatment difference (dB=dC=0), or to the alternative hypothesis of an expected true 0.15 benefit of treatment B (dB=0.15)

**Table 5:** Simulation results evaluating Rule 3 when the threshold probability is set at 0.90

Sample size	True benefit		Posterior mean estimate biases			Average sample sizes			% Early stopping	
	dB	dC	p_A_	p_B_	p_C_	A	B	C	B	C
40	−0.15	0.00	0.0092	0.0182	0.0254	39.9621	39.8136	37.9895	0.54 %	6.45 %
	−0.05	0.00	0.0088	0.0237	0.0272	39.8054	38.8511	37.8795	3.56 %	6.90 %
	**0.00**	**0.00**	**0.0085**	**0.0254**	**0.0270**	**39.6095**	**37.9435**	**37.9783**	**6.69 %**	**6.53 %**
	0.05	0.00	0.0089	0.0324	0.0250	39.5103	36.5882	38.0288	11.66 %	6.37 %
	0.10	0.00	0.0062	0.0384	0.0264	39.2740	34.3440	38.0311	19.85 %	6.37 %
	**0.15**	**0.00**	**0.0056**	**0.0416**	**0.0254**	**39.0452**	**31.9203**	**38.0610**	**29.96 %**	**6.22 %**
	0.20	0.00	0.0049	0.0451	0.0256	38.8153	28.6999	37.9511	42.93 %	6.61 %
	0.25	0.00	0.0051	0.0423	0.0254	38.5798	25.0534	37.9815	57.41 %	6.60 %
	0.30	0.00	0.0052	0.0369	0.0250	38.4489	21.2180	38.0583	71.85 %	6.33 %
	0.35	0.00	0.0037	0.0261	0.0270	38.2338	17.3342	37.9999	83.87 %	6.49 %
	0.40	0.00	0.0039	0.0098	0.0265	38.0706	14.1594	37.8966	92.38 %	6.79 %
	0.45	0.00	0.0035	−0.0115	0.0266	38.0738	11.1299	37.9628	97.64 %	6.54 %
100	−0.15	0.00	0.0029	0.0093	0.0226	99.8389	99.4843	94.1617	0.55 %	6.49 %
	−0.05	0.00	0.0031	0.0169	0.0237	99.3256	96.7377	93.7367	3.55 %	6.94 %
	**0.00**	**0.00**	**0.0020**	**0.0233**	**0.0222**	**99.0139**	**93.7839**	**94.1902**	**6.91 %**	**6.48 %**
	0.05	0.00	0.0013	0.0322	0.0224	98.3338	89.3276	94.1794	12.36 %	6.48 %
	0.10	0.00	0.0012	0.0405	0.0227	97.6239	82.2664	94.1533	21.90 %	6.50 %
	**0.15**	**0.00**	**−0.0009**	**0.0509**	**0.0234**	**96.5788**	**71.2643**	**94.0376**	**37.94 %**	**6.60 %**
	0.20	0.00	−0.0025	0.0578	0.0235	95.7497	56.8519	94.0065	59.48 %	6.66 %
	0.25	0.00	−0.0025	0.0577	0.0230	95.1262	41.9689	93.9749	79.99 %	6.71 %
	0.30	0.00	−0.0030	0.0473	0.0235	94.5634	29.7273	93.9678	92.99 %	6.71 %
	0.40	0.00	−0.0025	0.0119	0.0239	94.0066	15.1126	93.8206	99.84 %	6.79 %
	0.45	0.00	−0.0027	−0.0110	0.0227	94.2995	11.7989	94.1851	100.00 %	6.48 %

p_A_, p_B_ and p_C_ refer to the posterior means of response probability in arms A, B and C, respectively; δ*=0.15. Bold data refer to the null hypothesis of absence of any treatment difference (dB=dC=0), or to the alternative hypothesis of an expected true 0.15 benefit of treatment B (dB=0.15)

As expected, when the treatment was less efficacious than expected, the first rule allowed the trial to be stopped early in 30.7–52.5 % of cases when the absolute difference in response rates was 5 %, to 96–99 % of cases when the absolute difference was down to 20 % (Table 3). The mean sample size required to detect inefficacy was 25 patients for a decrease of 0.15 in response rates, down to 15 for a 0.20 decrease. Otherwise, the false negative stopping rates due to this first rule in the case of beneficial treatment were low, with values of approximately 15–23 % when there was no benefit, less than 10 % when the benefit was 5 %, and less than 1 % for higher benefits (Table 3).

To handle the control arm, rule 2 was then applied to detect the lack of treatment benefit (Table 4). Compared to the previous first rule, a decision of stopping early in case of actual lower response rates in the experimental group than in the control group appears to be reached similarly for small differences, with, for instance, a decision to stop in 32 % of cases compared to 31 % in the case of a 5 % response rate below that of the control for *n*=40 and in 46 % of cases compared to 52 % for *n*=100. In contrast, false negative decisions of dropping the arm were increased compared to rule 1 in the same situation; for instance, for a minor benefit of 5 %, the second rule incorrectly proposes stopping for futility in 13–16 % of cases compared to 7–9 % based on the first rule when *n*=40 and *n*=100, respectively. Expectedly, when ${\gamma}_2=0.10$, the results were modified, with lower false decision rates (Table 6).

**Table 6:** Simulation results evaluating Rule 2 when the threshold probability is set at 0.10

Sample size	True benefit		Posterior mean estimate biases			Enrolled sample sizes			% Early stopping	
	dB	dC	p_A_	p_B_	p_C_	A	B	C	B	C
40	−0.20	0.00	0.0625	0.0585	−0.0123	30.6959	13.8404	29.9078	92.23 %	34.59 %
	−0.15	0.00	0.0601	0.0311	−0.0120	31.2312	18.3044	29.5173	79.84 %	35.56 %
	−0.10	0.00	0.0567	0.0107	−0.0119	32.3427	22.4957	29.6238	63.89 %	35.43 %
	−0.05	0.00	0.0506	−0.0027	−0.0126	33.5772	26.5306	29.7194	48.34 %	35.68 %
	**0.00**	**0.00**	**0.0480**	**−0.0122**	**−0.0123**	**34.5352**	**29.3818**	**29.5852**	**35.89 %**	**35.74 %**
	0.05	0.00	0.0386	−0.0166	−0.0109	35.7946	32.2020	29.7191	25.14 %	35.03 %
	0.10	0.00	0.0337	−0.0178	−0.0107	36.7805	34.2756	29.8086	17.75 %	34.52 %
	0.15	0.00	0.0300	−0.0176	−0.0122	37.6091	35.9146	29.5542	12.22 %	35.64 %
	0.20	0.00	0.0268	−0.0180	−0.0111	38.1120	36.8978	29.8112	8.92 %	34.91 %
100	−0.20	0.00	0.0704	0.0581	−0.0229	66.7855	15.4632	65.8818	99.60 %	42.83 %
	−0.15	0.00	0.0673	0.0252	−0.0230	68.2876	23.4374	66.0635	96.28 %	42.43 %
	−0.10	0.00	0.0601	0.0007	−0.0229	71.7312	36.8674	66.5112	84.36 %	42.43 %
	−0.05	0.00	0.0571	−0.0160	−0.0231	75.3970	51.5977	65.8379	64.18 %	43.02 %
	**0.00**	**0.00**	**0.0467**	**−0.0237**	**−0.0240**	**81.2572**	**66.4872**	**65.8151**	**42.25 %**	**42.95 %**
	0.05	0.00	0.0379	−0.0255	−0.0233	86.5612	76.7592	66.9099	27.22 %	41.95 %
	0.10	0.00	0.0338	−0.0241	−0.0242	89.8261	83.4433	64.7309	18.46 %	43.92 %
	0.15	0.00	0.0279	−0.0229	−0.0232	92.3335	88.1690	66.3140	12.66 %	42.48 %
	0.20	0.00	0.0234	−0.0208	−0.0245	94.1323	91.5214	65.5106	8.98 %	43.34 %

p_A_, p_B_ and p_C_ refer to the posterior means of response probability in arms A, B and C, respectively; Δ=0. Bold data refer to the null hypothesis of absence of any treatment difference (dB=dC=0), or to the alternative hypothesis of an expected true 0.15 benefit of treatment B (dB=0.15)

Finally, when evaluating the third rule in detecting true benefits, the average sample sizes were decreased to about 10 patients per arm when the absolute benefit increased to 45 %, while the false positive rate was only 6–7 % in the case of no benefit, likely related to the threshold probability of ${\gamma}_3=0.90$ (Table 5). As expected, these figures were modified when using a less stringent probability threshold of ${\gamma}_3=0.80$ where the false positive rate reached 18–20 % in absence of any benefit (Table 7).

**Table 7:** Simulation results evaluating Rule 3 when the threshold probability is set at 0.80

Sample size	True benefit		Posterior mean estimate biases			Enrolled sample sizes			% Early stopping	
	dB	dC	pA	pB	pC	A	B	C	B	C
40	−0.15	0.00	0.0083	0.0320	0.0562	39.5748	38.6963	33.8148	3.64 %	18.64 %
	−0.05	0.00	0.0063	0.0495	0.0568	38.7270	35.8578	33.7310	12.09 %	18.95 %
	**0.00**	**0.00**	**0.0049**	**0.0555**	**0.0581**	**38.1052**	**33.9250**	**33.5867**	**18.38 %**	**19.20 %**
	0.05	0.00	0.0016	0.0632	0.0563	37.5141	31.3987	33.8662	26.92 %	18.51 %
	0.10	0.00	−0.0008	0.0691	0.0570	36.8763	28.0621	33.8537	38.65 %	18.55 %
	**0.15**	**0.00**	**0.0001**	**0.0720**	**0.0578**	**35.9576**	**24.3662**	**33.6137**	**51.87 %**	**19.22 %**
	0.20	0.00	−0.0002	0.0673	0.0561	35.5246	20.7723	33.8744	64.82 %	18.40 %
	0.25	0.00	−0.0017	0.0575	0.0568	34.9601	16.9213	33.7372	77.56 %	18.91 %
	0.30	0.00	−0.0043	0.0409	0.0575	34.3862	13.5320	33.6114	87.33 %	19.19 %
	0.35	0.00	−0.0016	0.0196	0.0560	34.5481	10.7458	34.0309	94.31 %	17.94 %
	0.40	0.00	−0.0033	−0.0069	0.0547	34.2171	8.5161	33.9205	97.80 %	18.43 %
	0.45	0.00	−0.0023	−0.0372	0.0542	34.1941	6.8534	33.9756	99.23 %	18.33 %
100	−0.05	0.00	0.0032	0.0229	0.0559	98.8583	96.4597	82.6701	3.69 %	18.83 %
	−0.05	0.00	−0.0006	0.0433	0.0563	96.1819	89.1834	82.5699	11.51 %	18.92 %
	**0.00**	**0.00**	**−0.0038**	**0.0577**	**0.0571**	**94.3563**	**82.2688**	**82.2694**	**19.21 %**	**19.14 %**
	0.05	0.00	−0.0060	0.0689	0.0570	92.3215	73.7509	82.5095	29.51 %	19.02 %
	0.10	0.00	−0.0075	0.0778	0.0567	89.5994	63.0008	82.5545	43.27 %	18.97 %
	**0.15**	**0.00**	**−0.0088**	**0.0822**	**0.0574**	**87.6736**	**49.5431**	**82.5278**	**61.69 %**	**18.88 %**
	0.20	0.00	−0.0102	0.0771	0.0530	86.2653	37.2856	83.2202	77.99 %	18.15 %
	0.25	0.00	−0.0113	0.0636	0.0558	84.2998	25.2895	82.5265	91.74 %	19.02 %
	0.30	0.00	−0.0112	0.0448	0.0576	83.4348	17.4124	82.3130	97.39 %	19.14 %
	0.35	0.00	−0.0114	0.0204	0.0558	83.0775	11.9089	82.5449	99.58 %	18.89 %
	0.40	0.00	−0.0102	−0.0073	0.0552	83.3131	9.0623	82.9624	99.95 %	18.47 %
	0.45	0.00	−0.0111	−0.0375	0.0585	82.5830	6.9659	82.3697	100.00 %	19.09 %

p_A_, p_B_ and p_C_ refer to the posterior means of response probability in arms A, B and C, respectively; δ*=0.15. Bold data refer to the null hypothesis of absence of any treatment difference (dB=dC=0), or to the alternative hypothesis of an expected true 0.15 benefit of treatment B (dB=0.15)

## Discussion

There has been increasing evidence that the effectiveness of clinical trials can be improved by adopting a more integrated model that increases flexibility and maximizes the use of accumulated knowledge. We focused this work on adaptive MAMS designs to select effective drugs among a fixed set of new drugs compared to a control. So-called screening or select/drop designs aim at proposing changes in treatment regimens with the possible elimination of a treatment group based on information derived from accumulated data. Such designs appear particularly useful for rapidly evolving interventions and drugs, especially when outcomes occur sufficiently soon to permit adaptation of the trial design. This setting in which several treatments are compared to a single control allows heterogeneity in patient populations and disease courses to be considered [24, 25]. However, the heterogeneity in objectives, design, data analysis, and reporting of these multi-arm randomized trials has recently been highlighted [26]. Moreover, in ascertaining which treatment modalities are most effective, the presence of K experimental arms also introduces complexity. We used a binary outcome measure, given that it appears to be the most widely used endpoint in phase II trials. Of note, such a binary criterion in MAMS has been used only in frequentist designs [6, 27].

Indeed, most of the proposed MAMS designs, including optimal designs, used a frequentist framework for inference [4–8, 14, 28]. The application of Bayesian adaptive design methods has recently been advocated to maximize the knowledge-creating opportunity of a learning phase study [13]. Surprisingly, although several designs have used Bayesian adaptive allocation methods[17, 29], Bayesian adaptive designs in terms of sample size or treatment allocation have been proposed mainly in the early phases of cancer drug development, notably in the setting of seamless phase I/II trials [13]. In the MAMS setting, Bayesian adaptive phase II screening designs have been proposed only for selecting/dropping arms using normal outcome measures [11], and more frequently by modifying the allocation probabilities to each arm. For instance, to select among treatment combinations of multiple agents, patients were adaptively allocated to either one of the treatment combinations based on posterior probabilities of all hypotheses of superiority of each combination based on a continuous endpoint [29]. Even when comparing MAMS designs to adaptive randomization designs, only the latter were based on Bayesian inference, whereas the former used test statistics from grouped sequential methods [27].

We decided to focus on the select/drop decisions while preserving the equilibrium of sample allocation across arms. We first use stopping rules based on the posterior probability of inefficacy (or of over-toxicity), as previously performed in closed settings [30, 31]. Indeed, nearly all phase III trials include pre-specified inefficacy/futility interim monitoring rules to stop the trial early if the interim results strongly suggest that the experimental treatment has no benefit over the control [32]. In contrast, a phase II analysis in a phase II/III trial requires more evidence that the experimental treatment works better than the control [2]. Thus, we use the difference of response probabilities between the treated group and control group as a simple Bayesian conditional measure of evidence regarding the treatment benefit. This method has been poorly used in a Bayesian context [12], possibly because the precise prior density of the difference of two independent beta is unknown. However, some analytical works have been published [18–20], and more recently, software to calculate the probability that one random variable is greater than another has been provided (http://biostatistics.mdanderson.org/SoftwareDownload/). When this density can be approximated, it can be used in several important applications. This illustrates how Bayesian methods give direct answers to the questions that most people want to ask, such as “which treatment is the best” [10]. Moreover, the Bayesian tools enable decision making based on the difference in response probabilities and the quantification of probabilities of benefit of each possible arm, which are more informative and transparent than *p*-values. It could be combined with the adaptive design methodology to provide a very flexible and efficient decision making process [33].

Due to the multiplicity of arms, we considered as the primary motivating design that of Xie et al. [13] who focused on multiple dose levels, though our approach was close to that proposed by Zalavsky et al. for tow-arm trials [12]. Nevertheless, this exemplifies the large interests and clinical applications of such Bayesian designs, unfortunately still underused in clinical practice [34].

Since a common concern in Bayesian data analysis is that an inappropriately informative prior may unduly influence posterior inferences, we reran the analyses using different priors, possibly distinguishing various amounts of previous information across randomized arms as quantified by the effective sample size. This slightly modified the results of the clinical trial. We restricted our considerations to conjugate beta priors so that the prior probabilities of tested hypotheses could be transformed into Bernoulli trials with a theoretical (effective) sample size. This appeared an important issue when applying Bayesian methods in settings with a small to moderate sample sizes such as those proposed for MAMS [21].

## Conclusions

Regardless of its inference, adaptive trial design is a methodologically sound way to improve clinical trials but adds significant complexity. This approach requires boundary parameters to be chosen for stopping decisions$.$ Xie et al. in 2012 [13] reported the use of a high criterion for action (${\gamma}_2$ =0.9) as a default value based on a maximum cohort size of 36 (with 24 treated with the active dose and 12 treated with placebo), although calibration is often required. Thus, we calibrated the values of these thresholds according to the simulation study. Indeed, the choice of these thresholds is highly dependent on our desire to control false decision in either direction, as typically considered in early trial phases. Otherwise, combining stopping rules 1 and 2 appears to be another option to improve such a control [33].

Finally, this adaptive Bayesian approach in which existing information at the time of trial initiation is combined with data accumulating during the trial has also been used to identify the treatments that are most beneficial for specific patient subgroups [35–38]. Such an approach, in the line of personalized medicine, appears to be an interesting research area to explore in the MAMS setting.

## Abbreviations

B(): Bernoulli distribution
Be(): beta distribution
ESS: effective sample size
MAMS: multi-arm multi-stage
MRT: minimum required treatment response
MSD: myelodysplastic syndrome
MSE: mean square error
mult(): multinomial distribution
STR: sufficient treatment response rate
